# Miscellaneous Intelligence

**Published:** 1820-04-01

**Authors:** 


### Miscellaneous Intelligence.

*Tic Douloureux.* The Case of Tic Douloureux, related in the last Number of our Journal, has procured us the honour of numerous suggestions relative to the treatment of the case: the following, though *anonymous*, we deem worthy of insertion.

"I have just read the case of the gentleman who is so *grievously* afflicted with "Tic Douloureux," and immediately on closing the perusal, have presumed to hint a mode of relief which I have found, in several cases, completely successful. I have generally observed, that the disease has been preceded, in its most severe attacks, by derangement of the *biliary*, and thereby of the *digestive* organs.

"On reading Dr. Marcet's paper, relative to the extract of strammonium, I immediately determined on the application of it to "Tic Douloureux," as a chance of alleviating, (to say the least) that most horrible disorder, and I must add that, in three cases, it has had the most *decisive* effect

"The method of *palliation* I shall say, pursued by me is as follows:—℞. Extracti strammonii gr. $\frac{1}{16}$th; pil. hydrarg. gr. ij; extracti colocynth. c. gr. ij M. Ft. 6tis horis sumend. and to avoid *stimulating* solids and fluids.

"The gradual relief obtained by the pill has been astonishing, and by the combination with the slight dose of colocynth, the blue pill has *never* acted on the mouth, nor has the strammonium produced the nausea which accompanies its use *alone*. If you think this letter worth insertion in your excellent work, it is much at your service, as my *sole* object in sending it, was the desire of advancing the utility of our profession."

Respecting the case in question, we are happy to say, that a great change for the better has already taken place, but we defer stating the means used, at present, till time shall have confirmed or blasted our hopes. REV.

P. S. We are much obliged to "POTEKA" for his valuable letter relative to the case in question.

---

Dr. NUTTALL, Physician to the Westminster General Dispensary, and a zealous cultivator of pathological and therapeutical knowledge, has formed an Arrangement of Diseases for the easy and effectual inculcation of practical medicine, in his Clinical Lectures at the above-mentioned Institution, an outline of which we here introduce, for the consideration of clinical teachers.

1. Each part is subject to morbid action, more or less extensive, in itself or its association with other parts of the body; with or without previous or subsequent organic injury.

2. All parts are subject to inflammation and change of structure with or without morbid action.

3. All the interstices and cavities are subject to dropsy; from want of balance between the sanguiferous and absorbent capillary system.

CLASS I. Diseases of the whole System.

| | | | |
|---|---|---|---|
| Idiopathic. | *Order* 1. Fever without specific eruption. Typhus, &c. | or | Without previous local injury. |
| | 2. Fevers with specific eruption. Variola, &c. | | |

Class II. Diseases of one or more of the three Fundamental Systems, generally complicated.

*Order* 1. Of the Sanguiferous System, *with Fever*.

Symptomatic. { Gen. 1. Fevers symptomatic of local inflammation.
2. Fevers symptomatic of suppuration. Hectic.

*Without Fever*,

Gen. 1. Irregular Circulation. Palpitatio. Congestio. Syncope.

| | | | |
|---|---|---|---|
| *Order* | 2. | Of the absorbent system, | Scrofula, &c. |
| | 3. | Of the nervous system, | Apoplexy, &c. |

Class III. Diseases of the Lesser or Subordinate Systems, frequently complicated.

| | | | |
|---|---|---|---|
| *Order* | 1. | Of the digestive system, | Dyspepsia, &c. |
| | 2. | Of the respiratory system, | Asthma, &c. |
| | 3. | Of the perspiratory system, | Ephidrosis, &c. |
| | 4. | Of the urinary system, | Diabetes, &c. |
| | 5. | Of the muscular and tendinous, | Tetanus, &c. |
| | 6. | Of the articular and bony, | Rachitis, &c. |
| | 7. | Of the sexual system. | Amenorrhœa, &c. |

Class IV. Diseases of Individual Organs.

| | | | | |
|---|---|---|---|---|
| *Order* | 1. | Of the sanguiferous, | Arteries, | Ossification, &c. |
| | 2. | Of the absorbent, | Glands, | Enlargement, &c. |
| | 3. | Of the nervous, | Brain, | Vesaniæ, &c. |
| | 4. | Of the digestive, | Stomach, | Pyrosis, &c. |
| | 5. | Of the respiratory, | Lungs, | Consolidation, &c. |
| | 6. | Of the perspiratory, | Skin, | Lepra, &c. |
| | 7. | Of the urinary, | Kidnies, | Nephralgia, &c. |
| | 8. | Of the muscles and tendons, | Muscles, | Extenuatio, &c. |
| | 9. | Of the joints and bones, | Bones, | Exostosis, &c. |
| | 10. | Of the sexual organs, | Vagina, | Gonorrhœa, &c. |
| | 11. | Of voice | | Aphonia. |
| | 12. | Of sight, | | Ophthalmia. |
| | 13. | Of smell, | | Epistaxis. |
| | 14. | Of hearing, | | Dysecæa. |
| | 15. | Of taste, | | Agheustia. |
| | 16. | Of touch, | | Anæsthesiæ. |

Though the Classes and Orders in this Arrangement are, from its nature, unalterable, the proper place of several genera is, in the present state of our knowledge, uncertain. On these points, particularly, I shall always feel happy to receive instruction.

---

*Palsy and Epilepsy*. We understand that Dr. Cooke's second volume on Nervous Disorders, embracing the subjects of Palsy and Epilepsy, is in considerable forwardness; and we are authorised to say, that he will thankfully receive any communications respecting those diseases, from his professional brethren, and acknowledge the same in the approaching volume; his object being to exhibit a concise exposition not only of the ancient, but of the modern opinions and practices relative to the above-mentioned disorders.—*Ed.*

Dr. CHARLES HASTINGS, Physician to the Worcester Infirmary, has in the Press, in one volume 8vo. a Treatise on Inflammation of the Mucous Membrane of the Lungs; to which is prefixed an Experimental Inquiry into the General Nature of Inflammation, and the Contractile Power of the Blood Vessels.

Mr. B. HUTCHINSON, Member of the Royal College of Surgeons, has in the Press Illustrations of Cases of Tic Douloureux successfully treated in a single volume.

*In the Press,* a Third Edition of Dr. MERRIMAN's Synopsis of the various kinds of difficult Parturition; with Additions and an Appendix of Illustrative Cases. Plates, &c.

---

### TO THE PUBLIC.

We have received several letters from our Subscribers, suggesting the adoption of an arrangment between the three *Quarterly* Medical Journals, by which they might be made to appear *successively* instead of simultaneously, as two of them now do. By the anticipation of one month in the next Number of this Journal, it will be so arranged that a Quarterly Medical Journal shall, in future, be published on the first day of every month. Thus the Quarterly Journal of Foreign Medicine and Surgery will appear on the 1st of May; the Medico-Chirurgical Journal, on the 1st of June; and the Edinburgh Medical and Surgical Journal, on the 1st of July, and so on in rotation. To those who take in but one periodical publication, this arrangement can be no inconvenience. To those who take in two, or more, it will prove a vast accommodation, as it divides the time, and keeps up a source of novelty and information.

It may also be observed, that these three Journals are very different, and almost distinct in their character and arrangement. One is solely confined to Foreign Medicine; the Edinburgh Journal is distinguished by the great extent and importance of its original Communications; while our own Journal is, in fact, an extended Analytical, Eclectic, and Critical Review, of all that is *practical and useful* in the medical literature of this country in particular. In adopting this arrangement, *we* can have no object but that of accommodating the Public. We request, then, that our Subscribers and Readers will be punctual in ordering this Work for the 1st of *June*, instead of July; and we hope to open the Third Volume with a Number of no common interest and importance.

It has given us much concern to receive, from various quarters, complaints that the Journal could not be procured with the other periodical publications at the beginning of the month in each quarter. This is the more vexatious, as we close the press work ten days before the publication, and deliver the work to the London Booksellers three or four days previous to the first of the month—which is not the case with any other Journal. Upon tracing several instances to their source, we found the cause to invariably lie with the country booksellers, who gave inaccurate titles of the work; confounding it with the Medical and Surgical Journal of Edinburgh, or the Medico-Chirurgical Transactions. The *technical* denomination of the work, in the *trade*, is, "Johnson's Medico"; but, to avoid all mistakes, it is requested, that Subscribers and Purchasers will cause their country booksellers to order distinctly from their London Correspondents—"Johnson's Medico-Chirurgical Review." The term *Review* will distinguish it from all other Journals, and, in fact, is the proper denomination of the work, as of late constructed.

We may also state, that the whole of the back Numbers, even the last Number published, are so nearly out of print, that we shall have no other alternative than that of either committing them all again to the press, or commencing the Third Volume as a New Series. We are not yet determined which method to adopt.

### EXTRA LIMITES.

Authors, and the profession in general, are respectfully informed, that although the established limits of the Journal shall be strictly preserved for *Analytical Reviews alone*, yet, as an accommodation to authors or others, who may be desirous of appealing to the public, on purely medical matters, whether in support of opinions or practices; in remonstrances against what they may conceive to be rigid or unjust criticism; or in temperate philosophical discussions, an *extra* space will, at any time, be allotted for the above-mentioned purposes, the author, at the same time, defraying the prime cost of paper and letter-press, as calculated by the printer of the Journal. When it is recollected, that the Editor derives from these *extra* portions, neither a saving of labour nor of expence, the aforesaid conditions cannot be considered illiberal. At a very trifling expence to individuals, the aggregate of which would be ruinous to the Journal, a facility is thus afforded to all whose inclinations prompt them to lay their sentiments before the profession at large. The only prerogative the Editor claims is, that of controlling any language or sentiments that he may deem detrimental to the harmony, or derogatory to the dignity of scientific discussions.

"Arcanum" will find that no Anonymous Reviews of puffing Publications shall ever find their way into this Journal.

APPENDIX.

